# The anticancer activity and mechanisms of She medicine herbs

**DOI:** 10.3389/fphar.2025.1610301

**Published:** 2025-07-17

**Authors:** Yunxuan Miao, Yisheng Chen, Qiaofen Lan, Ruogu Chen, Jiajia Zhuang, Haojun Shi, Miao Wang, Jianhui Miao, Chengshou Lin

**Affiliations:** ^1^ Henan University of Science and Technology, Luoyang, Henan, China; ^2^ Fujian Key Laboratory of Toxicant and Drug Toxicology, Medical College, Ningde Normal University, Ningde, Fujian, China; ^3^ Ningde Municipal Hospital of Ningde Normal University, Ningde, Fujian, China; ^4^ Fujian Medical University, Fuzhou, Fujian, China; ^5^ Animal Medicine, College of Life Sciences, Longyan University, Longyan, Fujian, China; ^6^ Faculty of Chinese Medicine and State Key Laboratory of Quality Research in Chinese Medicines, Macau University of Science and Technology, Macau, China; ^7^ Department of Orthopaedics, Mindong Hospital Affiliated to Fujian Medical University, Fuan, China

**Keywords:** She medicine, anticancer herbs, flavonoids, quercetin, cancer research

## Abstract

She Medicine, a traditional therapeutic system from China’s She ethnic group, shows promise in cancer treatment. This paper provides a comprehensive review of She medicinal herbs, focusing on their anticancer activities and underlying mechanisms. Compared to widely studied traditional medicines (e.g., Traditional Chinese Medicine), She Medicine exhibits unique ethnopharmacological traits, such as localized plant usage and multi-target mechanisms involving apoptosis induction, immune modulation, and tumor microenvironment regulation. Key herbs like *Pimpinella diversifolia* and *Melastoma dodecandrum* showing significant anticancer potential due to their bioactive compounds such as flavonoids, quercetin, and gallic acid. For example, homoharringtonine (HT), a She-derived alkaloid, targets Smad3/TGF-β pathways in non-small cell lung cancer and synergizes with chemotherapy in leukemia treatment, as evidenced by preliminary clinical trials. However, challenges persist, including resource shortages, insufficient mechanistic studies, and a lack of quality control standards. Future research should integrate multi-omics and bioengineering approaches to standardize She Medicine and bridge its traditional use with modern therapies such as immune checkpoint inhibitors. Overall, She medicinal herbs hold great promise for cancer treatment and warrant further exploration to unlock their full potential in modern medicine.

## 1 Introduction

The She people, one of China’s ancient ethnic minorities, primarily inhabit the mountainous regions of southeastern provinces, such as Fujian, Zhejiang, Guangdong, and Jiangxi (Liu et al., 2017; Chen, 2019). Jingning She Autonomous County in Lishui City, Zhejiang Province, as the only She Autonomous County in the country, serves as the core area for the inheritance of She Culture. She medicine emerged in the long process of the She people adapting to their natural environment and their relentless struggle for survival and health (Lin Y., 2023; Lin H., 2023). Unlike mainstream Traditional Chinese Medicine (TCM), She Medicine retains distinct ethnopharmacological practices, such as the use of alcohol-based remedies for rapid therapeutic penetration and localized herbs like Melastoma dodecandrum for gastrointestinal bleeding. The field of diagnosis and treatment in She Medicine is extensive, covering trauma, rheumatic pain, and infections (Rajath et al., 2024; Roshan et al., 2023; Folk-lore and Medicine, 1939). A 1999 survey of She practitioners revealed a focus on bone injuries (50%) and snake bites (17%), reflecting adaptations to local environmental challenges (Lei et al., 2014; Kim et al., 2016). She doctors also treat dysentery, syphilis, and eye diseases, demonstrating broad therapeutic scope (ДВ and ЛВ, 2023).

She medicine employs both medicinal and non-medicinal therapies. Medicinal treatments include oral and external applications, with innovations in dosage forms such as alcohol-based patches for arthritis (Li et al., 2015; Xue and O’Brien, 2003; Liu et al., 2019). While early She medicine relied on single-herb formulas, modern practices increasingly use compound formulations, though a unified theoretical framework remains underdeveloped (Lei et al., 2014; Gedif and Hahn, 2002; Sharma T. et al., 2022). The Asteraceae family dominates She Medicine, with 37 species used holistically, maximizing resource utility (Lei et al., 2014; Da Silva et al., 2023). This contrasts with TCM’s emphasis on roots and rhizomes, highlighting She’s ecological adaptation. Globally, rising cancer mortality underscores the urgency for innovative therapies. She Medicine’s unique bioactive compounds—such as homoharringtonine in leukemia and *Scutellaria baicalensis* flavonoids—offer multi-target mechanisms complementary to synthetic drugs (e.g., paclitaxel) and immune therapies. However, challenges like resource scarcity and insufficient clinical validation hinder its integration into modern oncology (Singh S et al., 2021; Sharma et al., 2024; Huang et al., 2024).

## 2 Anticancer potential of She medicine herbal drugs

### 2.1 Common anti-cancer She medicinal herbs

**FIGURE 1:**
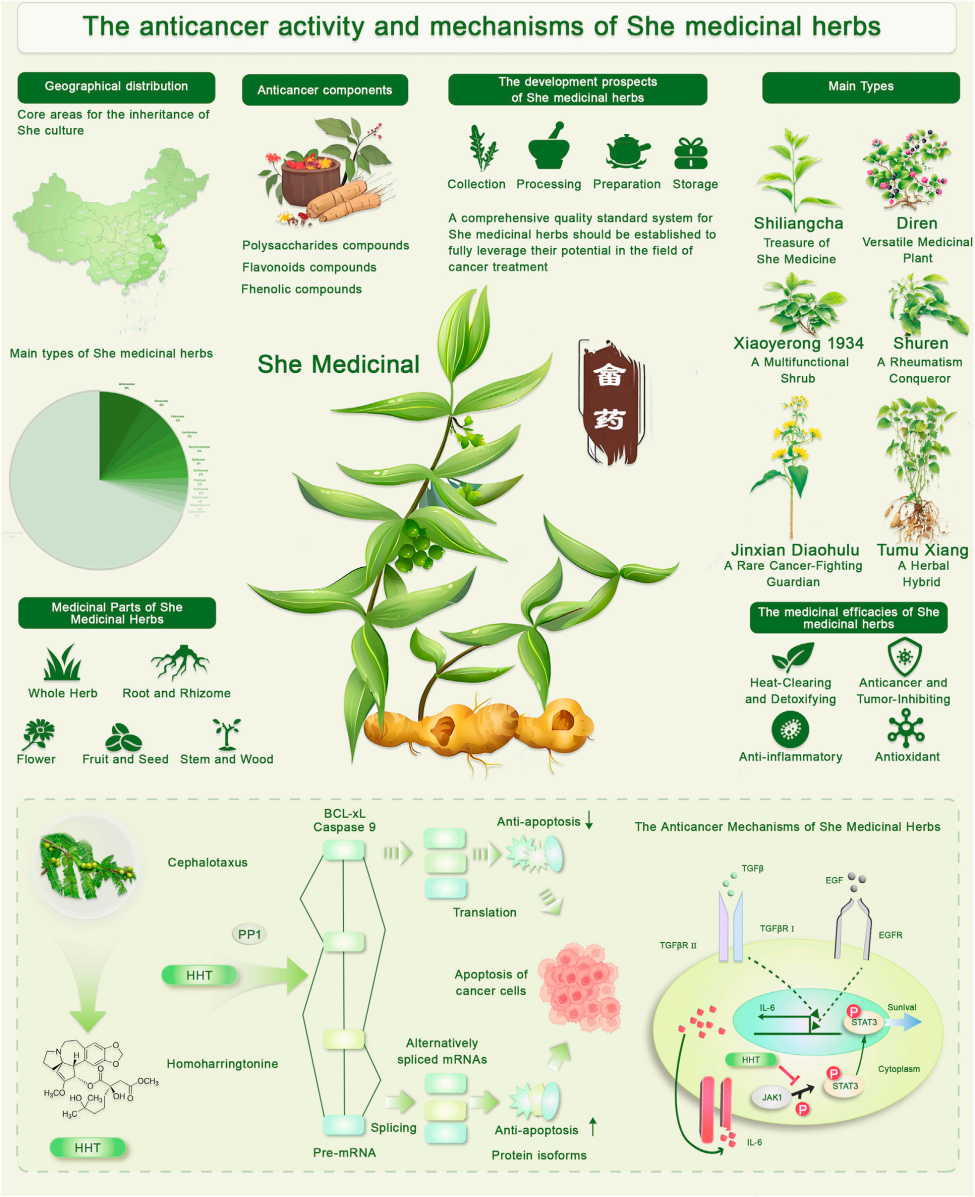
Anticancer activity and mechanisms of She medicinal herbs. The figure illustrates the anticancer activity and mechanisms of She medicinal herbs. It provides an overview of the medicinal components, therapeutic potential, and molecular mechanisms involved. The figure highlights the geographical distribution of She culture, which is centered in specific regions known for the use of these herbs. Key anticancer components, including polysaccharides, flavonoids, and phenolic compounds, are shown as vital bioactive substances within the herbs. The development of She medicinal herbs for cancer treatment is discussed, with an emphasis on the importance of standardized collection, processing, preparation, and storage methods to maximize their efficacy. Additionally, the figure lists major types of She medicinal herbs, such as Shiliangcha, Diren, Xiaoyerong 1934, Shuren, Jinxian Diaohulu, and Tumu Xiang, each noted for their unique medicinal properties. The medicinal parts of these herbs, such as the whole herb, root, flower, fruit, and stem, are also illustrated. Finally, the figure depicts the anticancer mechanisms of these herbs, highlighting the roles of compounds like HHT (Homoharringtonine) and Cephalotaxus in triggering apoptosis in cancer cells through various molecular pathways, including the regulation of BCL-XL, caspase 9, and the alternative splicing of mRNAs. This information underscores the potential of She medicinal herbs in cancer therapy.

Cold tea is one of the most commonly used traditional medicines among the She people. Modern studies have shown that the active ingredients of cold tea, in addition to volatile oils, include flavonoids, coumarins, anthraquinones, and other non-volatile compounds, with flavonoids being the dominant component. Modern pharmacological studies suggest that the anticancer effects of flavonoids in cold tea are closely linked to their ability to induce apoptosis and inhibit angiogenesis. In She medicine, cold tea is primarily used to treat conditions caused by food stagnation, such as fullness, chronic gastritis, gastric and duodenal ulcers leading to bloating, gastric pain, and acid reflux, as well as playing a crucial role in the prevention and treatment of colds and influenza (Liu et al., 2013; Samanta, 2022; Luo et al., 2024). Diren, derived from the whole plant *Melastomadodecandrum* (Lour.), is known for its sweet and astringent taste. Modern clinical studies have confirmed that Diren has notable hemostatic effects, particularly in treating gastrointestinal bleeding. Studies show that Diren is rich in polysaccharides, flavonoids, amino acids, phenols, and both macro and trace elements, contributing to its hemostatic, antioxidant, analgesic, anti-inflammatory, and hypoglycemic effects. Flavonoid compounds from Diren have demonstrated apoptosis-inducing and immune-modulatory properties, enhancing its potential as an adjunct therapy in gastrointestinal cancers. Further reports highlight its antitumor, hypoglycemic, and hypolipidemic properties, while showing no toxic side effects on normal cells (Zheng et al., 2021; Xie et al., 2016; Yao et al., 2021). Jinxian Diao Hulu: Many bioactive compounds, such as dandelion terpenes, flavonoids, and sterols, These compounds contribute to its anti-tumor, immune regulating, hepatoprotective, anti-inflammatory, and antiviral properties, and no toxicity has been observed. It is used clinically to treat febrile seizures, pneumonia, and various cancers in children (Gezici and Şekeroğlu, 2019; Chang et al., 2013). Xiaoye Rong 1934: a shrub commonly used in She medicine in Eastern and Southeastern China, is used to treat various conditions such as infantile malnutrition, indigestion, diarrhea, hernia, gout, and joint pain. The plant has been widely studied for its phytochemical components, and research has begun to explore its potential antitumor effects through pathways such as oxidative stress inhibition and immune modulation (Li et al., 2022; Lu et al., 2022). Tu Mu Xiang: Tu Mu Xiang, also known as “Hong Mu Xiang” or “Nan Wu Wei Zi Gen,” is a common herbal remedy among the She people of Fujian province. Research has shown that its source plants include multiple species from the Magnoliaceae family, particularly the roots and stems of *Schisandra chinensis*. These compounds exert anticancer activity by modulating apoptosis pathways and suppressing metastasis-related signaling (Yang et al., 2017; Zhang et al., 2022). Scutellaria baicalensis: Commonly used in She medicine for its multiple pharmacological effects, particularly anticancer properties. The key component *baicalin* has been shown to induce apoptosis in cancer cells, inhibit tumor angiogenesis, and reduce metastasis (Liu Y. et al., 2024). Panax notoginseng: Known for its effects on blood circulation and pain relief, it has demonstrated significant potential in anticancer therapy by inducing apoptosis and inhibiting the proliferation and invasion of cancer cells (Hawthorne et al., 2022). Lonicera japonica: Widely used for its anti-inflammatory and detoxifying properties, its active ingredients, such as chlorogenic acid and quercetin, show anticancer activity by inhibiting cell proliferation and inducing apoptosis (Ma et al., 2024; Park et al., 2017). Ganoderma lucidum: This popular herb enhances immunity and exhibits antioxidant, anti-inflammatory, and anticancer properties through its polysaccharide content (Cör et al., 2018; Sohretoglu and Huang, 2018). Hedyotis diffusa: An important part of She medicine, its active ingredients are widely used for their anti-cancer, anti-inflammatory, and anti-oxidant effects, particularly in the treatment of liver and stomach cancers (Figure 1) (Shao et al., 2011).

In comparison with conventional therapies such as paclitaxel or platinum-based chemotherapy, these She medicinal herbs provide multi-targeted mechanisms—including apoptosis regulation, immune modulation, and microenvironment adjustment—highlighting their potential as complementary or alternative options in integrative cancer therapy.

### 2.2 Main medicinal parts and efficacy of She medicine anti-cancer herbs

From the perspective of medicinal parts, Asteraceae family herbs in She Medicine most frequently utilize the whole plant with 37 species falling into this category, followed by root and rhizome, flower, fruit and seed, and stem wood types (Sharma M. et al., 2022). There are 20 species with two or more medicinal parts, including *Artemisia lactiflora* Wall. ex DC. and *Eupatorium japonicum* Thunb. This reflects the traditional emphasis on maximizing pharmacological utility by using multiple plant parts (Lei et al., 2014; Yarnell, 2015). For example, *Rubus chingii* (Eastern raspberry) has dry roots that are used in She medicine to treat wind pain, improve vision, expel toxins, and soothe nausea. It is applied for treating conditions such as tuberculosis and spinal cord compression (Sheng et al., 2020; Yu et al., 2019) *Di Ren*: A commonly used herb in She medicine, the whole plant is used. It is sweet and slightly cool, containing polysaccharides and flavonoids. It has hemostatic, antioxidant, and anti-inflammatory properties. The highest concentration of active compounds is found in the leaves (Sze et al., 2010). *Shi Liang Cha*: Derived from the dry leaves of *Lyonia* species, it has a cooling, slightly bitter, and pungent nature. It is used in treating wind-heat symptoms, with significant antioxidant and anti-tumor effects. It is clinically applied for diseases like colds and diarrhea (Ma et al., 2017). *Di Jin Ju*: The whole plant is used and is beneficial for activating blood flow and expelling wind, and it treats gastric pain. This herb has been used in She communities for generations (Wali et al., 2022). The use of whole plants or multiple parts enhances the synergistic efficacy of She Medicine. This holistic approach differs from modern single-compound therapies by preserving the complex phytochemical interactions essential for therapeutic effects.

## 3 Anticancer activity and mechanisms of She medicine herbs

### 3.1 Active ingredients and their biological activities

Flavonoids, triterpenes, alkaloids, and polyphenols found in She medicine exhibit a wide range of biological functions, including anticancer properties. For example, flavonoids in Shiliang tea may be the basis of its cardiovascular protection, antibacterial, antiviral and anticancer activities (Wang et al., 2020; Li and Jiang, 2018). Anthraquinone compounds such as rhein methyl ether and rhein-8-O-β-D-glucoside contribute to antibacterial, anti-inflammatory, and potentially cytotoxic effects in cancer cells. Harringtonine (HT), a natural alkaloid from Cephalotaxus, has shown strong efficacy against leukemia and is currently used in the treatment of acute leukemia and lymphoma (Luo et al., 2004). The study of HT derivatives showed that ht1 enhanced the antiproliferative activity of HL-60 cells, with an IC_50_ value approximately 2,000 times lower than that of HT itself (Ochi et al., 2022; Sakamoto et al., 2018). These results highlight the importance of chemical modification in enhancing bioactivity. In Sanjiaofeng dew, extraction efficiency of active ingredients like flavonoids is influenced by solid-liquid ratios and microwave irradiation time, which are critical factors in maximizing anticancer efficacy during preparation. These components induce tumor cell apoptosis and inhibit proliferation by modulating key signaling pathways (Abotaleb et al., 2018; Raina et al., 2020).

### 3.2 Progress in chemical constituents and anticancer research

In recent years, notable advances have been made in the phytochemical investigation of She medicine herbs. For example, the flavonoids in the extract of Liquidambar sanjiaoensis are essential for anti-cancer and immune regulation (Luo et al., 2004). The pharmacological effects of Di Ren and San Jiao Feng Lu are directly related to the contents of flavonoids and phenols. By analyzing the content of gallic acid and quercetin in different parts of Rehmannia glutinosa, the researchers determined the best harvest time of Rehmannia glutinosa (Yue-ling et al., 2017). Its extracts, rich in quercetin and gallic acid, show significant variability across plant parts and seasons, affecting therapeutic efficacy (Engström et al., 2015). Quercetin, a key flavonoid, reduces oxidative stress and inhibits tumor cell proliferation through cell cycle arrest (Xie et al., 2011). Its concentration reached its peak in early May, making it a critical compound for maximizing anticancer activity during harvesting (Xie et al., 2012).

### 3.3 Extraction and analysis of active ingredients

Many herbs in she medicine contain bioactive compounds such as flavonoids, saponins, quinones and terpenes, considered core contributors to their anticancer activity^198,199^. For example, astragaloside IV from Astragalus enhances immune responses and suppresses tumor progression (Zhou et al., 2017). The chemical constituents of Euphorbia officinalis, including flavonoids and quercetin, have been proved to inhibit the proliferation of tumor cells (Engström et al., 2015; Yang et al., 2021). Quercetin not only regulates the cell cycle but also significantly increases the apoptosis rate of malignant cells. Researchers have employed advanced analytical techniques such as high-performance liquid chromatography (HPLC) and gas chromatography–mass spectrometry (GC-MS) to identify and quantify bioactive ingredients in Camellia and other She herbs. Emerging tools including single-cell sequencing, network pharmacology, and molecular docking are increasingly used to uncover the intricate anticancer mechanisms of She herbal medicines (Huang et al., 2024; Qi and Zou, 2023; Ni et al., 2025; Jiao et al., 2021).

### 3.4 Anticancer mechanism

**FIGURE 2:**
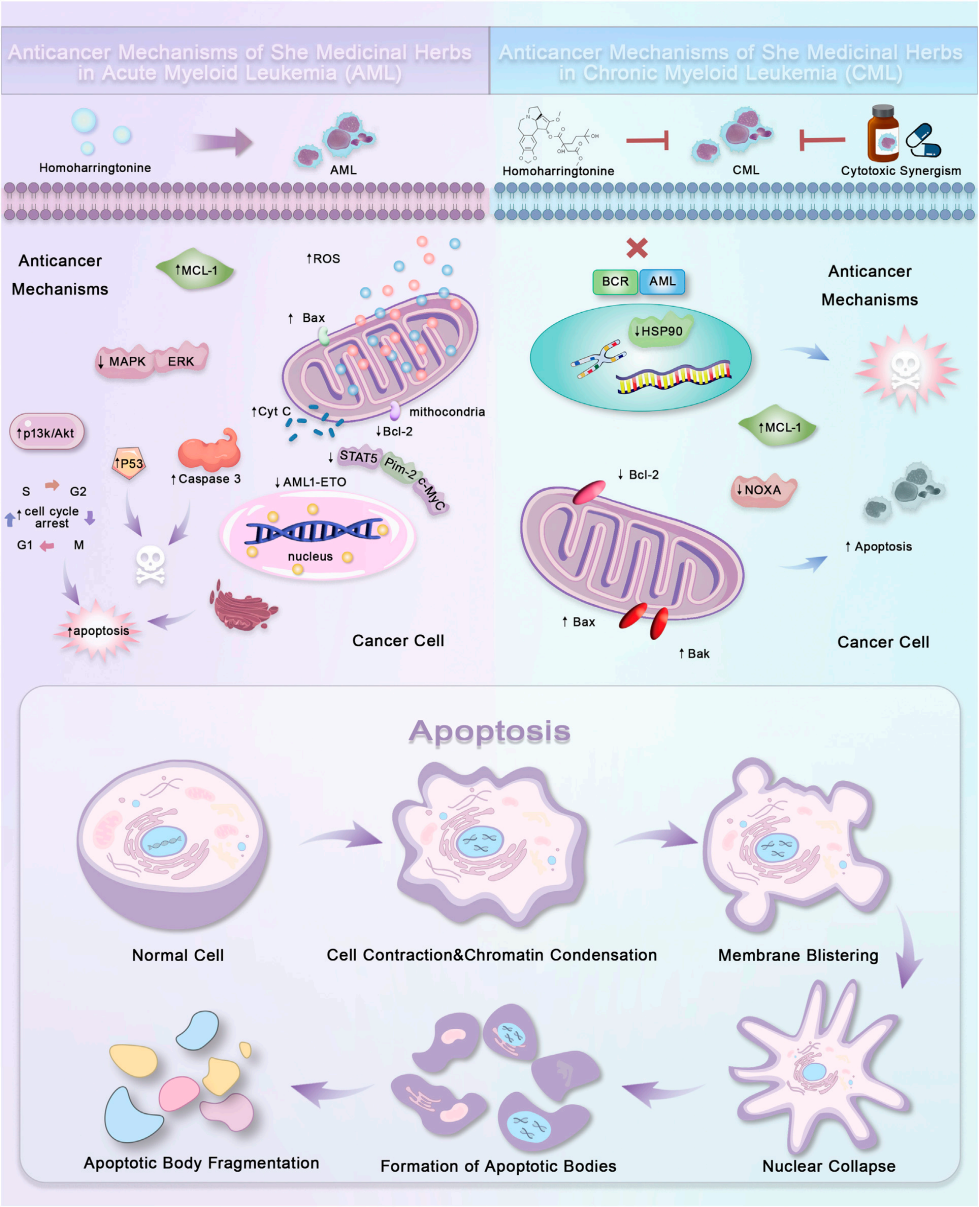
Anticancer mechanisms of She medicinal herbs in acute and chronic myeloid leukemia. The figure illustrates the anticancer mechanisms of She medicinal herbs in the treatment of Acute Myeloid Leukemia (AML) and Chronic Myeloid Leukemia (CML). In AML, Homoharringtonine induces apoptosis by upregulating MCL-1 and ROS, activating Bax, and promoting mitochondrial release of cytochrome c, which triggers a cascade of apoptotic events through p53 activation, caspase 3 and 9, and cell cycle arrest. In CML, Homoharringtonine works in synergy with cytotoxic agents to inhibit BCR-ABL fusion proteins, modulate HSP90, and downregulate Bcl-2 and MCL-1, leading to Bax, Bak, and NOXA activation and apoptosis. The figure also shows the typical apoptotic morphological changes, including cell contraction, chromatin condensation, membrane blistering, apoptotic body fragmentation, and nuclear collapse, which occur during the cancer cell death process. These findings underscore the therapeutic potential of She medicinal herbs in leukemia treatment by targeting key apoptotic pathways.

At the cellular level, the components of She medicine can induce tumor cell apoptosis by modulating apoptosis-related genes and activating intrinsic and extrinsic apoptotic pathways (Yang et al., 2023a; Yang et al., 2023b). For example, triptolide activates caspase enzymes and suppresses Bcl-2 expression to initiate programmed cell death (Davis and Salton, 1975; Safarzadeh et al., 2014; Lefkowitz, 1975). In AML, homoquercetin upregulates MCL-1 and ROS levels, activates Bax, and promotes mitochondrial release of cytochrome c to trigger apoptosis (Figure 2). At the molecular level, HT showed anti-cancer effect on gefitinib resistant NSCLC cell lines by targeting Smad3 and TGF-β signaling pathways. It also regulates alternative splicing of Bcl-x and caspase-9 through a PP1-dependent mechanism (Cao et al., 2015; Qin et al., 2015; Wang et al., 2021; Gao et al., 2007). At the tissue level, She medicine inhibits tumor angiogenesis, invasion, and metastasis. This is achieved through comprehensive regulation of the tumor microenvironment, such as downregulating pro-inflammatory cytokines and restoring intestinal microbiota balance (Yang W. et al., 2023; Liang et al., 2024; Zhou et al., 2023a). Immunocytochemical and pathway studies have shown that She medicines enhance immune surveillance and counteract tumor immune evasion by modulating the infiltration of tumor-associated macrophages and T-cell subtypes. They also inhibit key pathways like NF-κB, offering mechanistic insights into their immunomodulatory effects (Ding et al., 2025). Some She medicines, such as Astragalus and Codonopsis, stimulate macrophage and T cell activity, amplifying antitumor immune responses (Patanapongpibul and Chen, 2019). With the integration of single-cell transcriptomics, bioinformatics, and experimental validation, researchers can now delineate She medicine’s impact on immune cell subtypes within the tumor microenvironment, providing robust evidence for its immunotherapeutic value (Yang et al., 2023b; Liang et al., 2024; Qian et al., 2024; Yu and Pan, 2024; Luo et al., 2023).

### 3.5 Related research and technological innovation

Genetic analysis of ITS2 sequences in raspberry and related species revealed distinct molecular markers, offering new insights into Rubus taxonomy and the evolutionary basis of anticancer activity in She herbal species (Song et al., 2012). A new she medicine crushing device was designed to improve processing efficiency and standardization, ensuring uniform quality for research and clinical use (Thapliyal et al., 2024). The device enhances purity and consistency, which are essential for reproducibility in pharmacological studies. Simultaneously, bioactive scaffolds are being used to deliver She herbal extracts to tumor tissues with enhanced targeting and controlled release, forming a novel strategy for She medicine-based anticancer drug delivery systems (Jia et al., 2024; Li et al., 2024). In summary, She medicine presents a promising approach to cancer therapy through its chemically diverse components and multi-target mechanisms.

## 4 Clinical applications and case studies

### 4.1 Combination chemotherapy and radiotherapy

Numerous clinical studies suggest that She medicinal herbs can be used as adjuncts to chemotherapy drugs to improve their efficacy (Wang et al., 2012; Wang et al., 2018; Murasawa et al., 2023). For instance, extracts from Bai Ji (Radix Bletillae) and *Tripterygium wilfordii* L. have been shown to significantly enhance the efficacy of standard chemotherapeutic regimens while concurrently reducing drug resistance and toxicity (Zhou et al., 2023b). In advanced cancer patients, She medicine is often employed to alleviate cancer-related symptoms, such as pain and fatigue, and to improve appetite and sleep quality, ultimately enhancing patients’ overall quality of life (Mystakidou et al., 2007). The use of golden-thread gourds (*Cocculus trilobus*) in She folk medicine has demonstrated empirical effectiveness in tumor management. Recent studies conducted by Chinese research institutions have confirmed its anticancer activity, particularly by inducing apoptosis in liver cancer and leukemia cell lines (Mystakidou et al., 2007; Dai et al., 2013). Mechanistic investigations suggest involvement of caspase-3 activation and modulation of mitochondrial pathways, indicating that this herb holds promise as a supplementary treatment. These findings align with a growing body of literature that supports the integrative use of traditional medicine in reducing chemotherapy side effects and enhancing clinical response.

### 4.2 Classic cases

In traditional She medicine, many She medicinal herbs are widely used not only for cancer but also for a variety of other chronic and inflammatory conditions. One notable example is the externally applied formula “She Medicine Twelve-Hour Ointment,” which exemplifies the distinctive therapeutic approach of She ethnomedicine and offers insights for translational applications in modern integrative medicine (Yu et al., 2016). Research has shown that the ointment possesses favorable stability and reproducibility during preparation, and exhibits significant analgesic and anti-inflammatory activity. The clinical application of this ointment in musculoskeletal pain, soft tissue injuries, and even localized cancer-related pain syndromes underscores the need for further exploration into topical She herbal therapeutics.

## 5 Challenges and limitations in the anti-cancer research of She medicine herbal drugs

While She medicine exhibits promising therapeutic potential in oncology, several critical limitations hinder its further development and clinical translation. Compared with other ethnic medicinal systems in China, She medicine research has progressed relatively slowly. For example, despite the identification of active components in the fruits and seeds of Schisandra sphenanthera, research on its roots and stems remains insufficient (Huang et al., 2021). This gap underscores the need for comprehensive, plant-part-specific analyses using modern tools such as LC-MS/MS and multi-omics approaches. The absence of standardized quality control frameworks is another major barrier. Since most She herbal drugs are sourced from the wild, there is considerable batch-to-batch variability in chemical composition, which can directly affect clinical efficacy and reproducibility (Li et al., 2013).

Additionally, soil pollution and the intrinsic genetic variability of medicinal plants may result in heavy metal accumulation and introduce safety concerns. Current studies reveal regional differences in She medicine safety profiles, attributable to ecological, climatic, and processing disparities. These would help analyze adverse event variability, and provide robust data to support the development of unified safety standards, risk control measures, and regulatory guidelines (Nyame et al., 2024). Establishing a comprehensive quality assurance system that encompasses cultivation practices, harvesting protocols, processing techniques, and formulation standards is essential for promoting the safe and standardized clinical application of She medicine in cancer therapy.

## 6 The future direction of She medicine in cancer research

She medicine represents a promising frontier in integrative oncology, offering multi-target, plant-based therapeutic strategies that complement modern cancer treatments. To fully harness its potential, future research should focus on comprehensive investigations into its active compounds and mechanisms of action through advanced methodologies such as single-cell RNA sequencing, multi-omics analysis, and spatial transcriptomics (Luo et al., 2023; Li G. et al., 2023; Zhu et al., 2022). Although preliminary studies have identified a range of bioactive flavonoids, alkaloids, and terpenes, their precise pharmacodynamics and pharmacokinetics remain incompletely understood.

Standardized extraction protocols, quality control frameworks, and pharmacological validation are essential prerequisites for clinical application. AI-based analytical tools, including machine learning algorithms and predictive pharmacology models, will facilitate the screening and prioritization of effective compounds, while optimizing extraction processes and formulation development (Ye et al., 2023; Li W. et al., 2023).

To evaluate anticancer efficacy, preclinical models such as orthotopic liver, breast, and gastric cancer animal models should be employed. These models allow for the identification of biomarkers responsive to She medicine and support subsequent validation in clinical studies (Qin et al., 2023; Ramirez-Hernandez et al., 2024). High-throughput screening and Box–Behnken response surface methodology have already proven useful in optimizing prescriptions, as demonstrated in the case of the Snake Medicine Twelve-Hour Ointment (Liu DSK. et al., 2024; Zhang et al., 2021).

The integration of she medicine with modern drug discovery platforms holds transformative potential. For instance, the traditional use of Taxus plants by She communities has stimulated the development of anti-leukemic agents. Current studies are investigating their metabolic pathways, biosynthesis, and target-specific interactions (Shao et al., 2021). Computer-aided drug design (CADD), molecular docking, and molecular dynamics simulations are being applied to structurally optimize She herbal compounds and predict their interactions with tumor-associated targets, thereby accelerating the discovery of low-toxicity derivatives (Nascimento et al., 2022).

Furthermore, targeted drug delivery systems such as flavonoid-loaded nanoparticles, liposomes, and ADCs are being developed to improve She medicine’s bioavailability, targeting accuracy, and safety profile. These technologies support the clinical translation of She herbal compounds and facilitate the transition from laboratory to bedside (Zhu et al., 2024; Rajendran et al., 2025; Zhu et al., 2025; Patra et al., 2018; Rajendran et al., 2025; Soroudi et al., 2024; Xi et al., 2024). A population-based cohort study has been conducted to assess biomarker changes before and after She medicine treatment, and meta-analyses are being employed to explore patterns of disease progression and response (Wu et al., 2024).

## 7 Conclusion

As a vital component of traditional Chinese medicine, She herbal medicine holds increasing importance in the field of cancer research and treatment. It not only draws from a rich foundation of ethnopharmacological knowledge, but also contains a diverse range of bioactive compounds with demonstrated anticancer potential. Through multi-target mechanisms—such as inhibiting cell proliferation, inducing apoptosis, modulating immune responses, and suppressing angiogenesis—She medicine provides a novel and holistic approach to anticancer drug discovery. However, several key limitations remain. The identification and characterization of active ingredients require deeper investigation, and the lack of standardized quality control protocols continues to hinder clinical translation. Future research should leverage advanced biotechnological platforms, including multi-omics, AI-assisted compound screening, and molecular pharmacology, to clarify mechanisms of action and identify therapeutic targets. Moreover, the efficacy and safety of She medicinal herbs must be rigorously validated through preclinical animal models and well-designed clinical trials.
